# Use of and Medical Decision-Making in Portal Messages Among Patients With Type 2 Diabetes: Mixed Methods Study

**DOI:** 10.2196/79413

**Published:** 2025-09-25

**Authors:** Terrence Liu, Eric A Waselewski, Ailish Dougherty, James D Lee, Nina E Hill, Brianna A Marzolf, Tammy Chang

**Affiliations:** 1 Department of Internal Medicine, University of Michigan Medical School, Ann Arbor, MI, United States; 2 VA Center for Clinical Management Research, Ann Arbor, MI, United States; 3 Department of Internal Medicine, University of Utah School of Medicine, 30 North Mario Capecchi Dr, Salt Lake City, UT, 84112, United States, 801-581-7822; 4 University of Michigan Medical School, Ann Arbor, MI, United States; 5 Department of Family and Community Medicine, University of Cincinnati College of Medicine, Cincinnati, OH, United States; 6 Department of Family Medicine, University of Michigan Medical School, Ann Arbor, MI, United States

**Keywords:** patient portal, medical decision making, type 2 diabetes, telehealth, secure message

## Abstract

We used a mixed-methods approach to characterize and understand the content of secure messages exchanges between patients with type 2 diabetes and their healthcare teams.

## Introduction

Patient portal messages allow individuals to directly communicate with their health care team. Portal messages are helpful for patients with type 2 diabetes (T2D) to receive important medical advice for managing T2D, including medication adjustments and diagnostic testing [1]. Portal messages involving medical decision-making can substitute for in-person medical visits and enhance access [2]. Yet, the types of medical decisions for T2D occurring through portal messages are poorly understood, possibly leading to low-value and inappropriate use of portal messaging by both patients and clinicians, such as messaging a physician regarding a medical emergency [3]. Improved understanding of medical decision-making is needed to inform the optimal use of this technology for T2D care, particularly using approaches that combine quantitative electronic health record (EHR) data and qualitative data from portal message texts. This study described medical decision-making occurring over portal messages between patients with T2D and their health care teams.

## Methods

### Ethical Considerations

This study was deemed exempt by The University of Michigan Institutional Review Board (HUM00245390). Informed consent was not needed because this was a retrospective study of large-cohort data. The data were only accessible to investigators. Participants were not compensated.

### Overview

We used EHR data to identify adults aged ≥18 with a diagnosis of T2D, defined as having a hemoglobin A_1c_ ≥6.5%, who received care from primary care practices within a large academic medical center from January 1, 2023 to December 31, 2023. Individuals were included if they initiated at least one portal message encounter and were prescribed diabetes-specific medication during the study period (Multimedia Appendix 1). A portal message encounter was the complete thread of messages initiated by the patient and responses by members of their health care team.

Using an explanatory sequential mixed methods approach [4], we quantitatively described demographic characteristics of our study cohort, use of portal message encounters, and characteristics of all patient-initiated portal messages encounters (number of messages per encounter, associated billing and ICD-10 codes; Multimedia Appendix 1).

To identify medical decision-making, we used national practice guidelines for diabetes management [5] and only coded billed message encounters, which require at least 5 minutes of clinician time for medical decision-making [6]. After identifying billed message encounters, we developed a codebook through qualitative content analysis of portal message text content [7,8]. All message encounters, including both patient and health care team member messages, were coded independently by two study team members (TL, EW, AD, JL, NH) with discrepancies resolved through consensus [9,10]. Summary statistics of demographic data and code frequencies were calculated using Stata statistical software (version 18; StataCorp, LLC). Microsoft Excel was used for qualitative coding.

## Results

Overall, 7653 individuals (3972 [51.9%] men, 3681 [48.1%] women; mean age, 62.9 [SD 14.5] y) were included with a median of 4 (IQR 2‐8) portal message encounters per individual (Table 1).

**Table 1.** Demographic and health characteristics of patients by portal message utilization level.

Characteristic	Utilization level tertile (n=7653)		
	Low (n=3492 [45.6%])	Medium (n=2012 [26.3%])	High (n=2149 [28.1%])
Total portal message encounters per patient, median (IQR)	2 (1-3)	5 (4-6)	12 (10‐19)
Age, mean (SD), years	62 (15)	63 (15)	64 (14)
Sex, n (%)			
Male	1934 (55.4)	1024 (50.9)	1014 (47.2)
Female	1558 (44.6)	988 (49.1)	1135 (52.8)
Race and Ethnicity, n (%)			
Asian or Native Hawaiian or Other Pacific Islander	299 (8.6)	139 (6.9)	100 (4.7)
Black or African American	429 (12.3)	218 (10.8)	196 (9.1)
Hispanic	102 (2.9)	59 (2.9)	59 (2.7)
Multi-race	94 (2.7)	37 (1.8)	50 (2.3)
White or Caucasian	2459 (70.5)	1504 (74.8)	1679 (78.2)
Other race or ethnicity^a^	109 (3.1)	55 (2.7)	65 (3.0)
Poorly controlled diabetes (hemoglobin A_1c_ >9%), n (%)	508 (14.5)	234 (11.6)	216 (10.1)
Having an insulin prescription, n (%)	1762 (50.5)	1157 (57.5)	1556 (72.4)
Billed portal message encounters, n (%)			
0	3358 (96.2)	1830 (91.0)	1721 (80.1)
≥1	134 (3.8)	182 (9.0)	428 (19.9)

a Other race or ethnicity category includes American Indian and Alaska Native, Middle Eastern or North African, individuals who selected “Other”, or individuals without race and ethnicity information.

There were 51,160 message encounters, with a median of 2 (IQR 2‐4) messages per encounter. Of all portal message encounters, 941 (1.8%) were billed, with billed encounters having a higher median number of messages per encounter compared to non-billed encounters (4 [IQR 2‐6] vs 2 [IQR 2‐4]).

Among all billed message encounters, recommendations from the health care team involving adjustments or changes to patient medications was the most common content category (“Pharmacologic Recommendation,” 682 encounters [73%]), followed by patient-initiated questions related to their medications (“Medication Questions,” 604 encounters [64.7%]), then medical recommendations from the health care team not involving medications (“Non-Pharmacologic Counseling,” 573 encounters [61.4%]; (Table 2). Of billed message encounters, 240 (25.5%) were linked with a T2D diagnosis code.

**Table 2.** Content and representative quotations of billed portal message encounters.

Message content code	Description of content code	Portal message encounters, n (%)^a^	Example representative quote
Patient-generated health information			
Subjective	Health information that the patient reports on their own health (pain, nausea, breathing issues, or diarrhea)	526 (56.3)	*“For the past two days my heart has been fluttering and I become short of breath and tired. It last for about two to three minutes. Should I go to the hospital or monitor it for another day or two?”*
Objective	Health information that the patient reports that is measured by a device (blood pressure, blood glucose, weight, and temperature)	259 (27.7)	*“Hi last night I checked my blood sugar manually with the meter and it was 150 so I calculated it on my receiver which was showing 40 and 50 readings. Calibrated I mean. So should I still decrease the insulin to Lantus 60 and Humalog 18. Right now it’s 139. On my receiver. I don’t want to have high blood sugars. Not really sure what to do. Sorry”*
Patient-initiated medical questions			
Diagnostic testing	Questions about testing, request for testing, choosing among testing options, why a test is important, or preparing for a test	154 (16.5)	*“Please order blood work including hormonal levels, A_1c_ for me before my scheduled appointment with you on [date].”*
Results of testing	Requests for discussion and the interpretation of diagnostic test results ordered by the health care team	163 (17.5)	*“I have a question about XR CHEST PA AND LATERAL resulted on [date]. I don’t understand the results. Is the pneumonia gone? Are there other problems?”*
Medication questions	Questions about medications, including efficacy, side effects, administration, dosing, renewals, and refills	604 (64.7)	*“I would like to stop taking Victoza. I have been experiencing nausea, diarrhea and constipation. Some days I feel great but more often I just don’t feel well. I thought over time these side effects would stop but they have not. What should I increase my insulin to? I am maintaining my same weight and diet, so I have not benefited in that regard. What are your thoughts?”*
Scheduling and referrals	Request to schedule a medical appointment or referral	153 (16.4)	*“Can you please tell me an ent doctor I need to see as left ear is getting duller or whatever way you call it. So I would like to go see one . And a podiatrist too. Big callus under my right foot. Than you.”*
Medical recommendations from the health care team			
Pharmacologic recommendation	Decision to start a new medications or management plan for an existing medication	682 (73.0)	*“You can increase your insulin to 7 units at night and 7 units with each meal*. *If you have a low blood sugar you can take 4 glucose tabs and recheck in 15 minutes*. *Call our office if you have a low blood sugar*. *Message me tomorrow with an update.”*
Referral to other clinicians	Referral to a different medical team than the responding team, including medical specialists or mental health providers (cardiologist, surgeon, psychiatrist, or psychologist)	151 (16.2)	*“Sorry to hear about that. I am glad that the bupropion offered at least a little benefit. If you are feeling such severe anxiety and stress that you are unable to work, I would recommend that you establish with both a psychiatrist and therapist”*
Referral to skilled therapy	Referral to a skilled therapist (physical therapist, occupational therapist, nutrition specialist, or dietitian)	50 (5.4)	*“I do not think we should repeat shoulder injection for finger pain. Does not make sense, esp if your shoulder is not currently painful*. *Are you wearing splint from OT? Please keep those appointments so we can see if any of the OT suggestions are helpful.”*
Non-pharmacologic counseling	Medical recommendations that do not involve management of medications (behavioral and lifestyle changes)	573 (61.4)	*“Your blood work would suggest that you have some sort of illness going on, perhaps a stomach bug (known as gastroenteritis). Are you having diarrhea, vomiting, abdominal pain, and/or fevers?* *If your symptoms are preventing you from keeping down food or water, you should seek medical attention*. *If you are keeping food and water down, but your symptoms are not improving, please contact the clinic and we can set up an appointment.”*
Diagnostic testing	Decision to obtain additional diagnostic testing	259 (27.7)	*“We can do an x-ray, but then it can get complicated figuring out what to do about it. I’ll order an x-ray, but you may still want to come in to the office for a real visit afterward.”*
Administrative^b^	Administrative issues that were discussed by either the provider or health care team (completion of paperwork for medical leave, health insurance coverage questions, prior authorization issues, financial questions related to paying for health care services, comments of frustration or appreciation)	221 (22.6)	*Provider: “I have placed your concern in a letter and sent it to you in your portal. I cannot write a letter to get someone out of jury dury but I can document your health conditions and symptoms to support an excuse.”*
Other^b^	Content not captured by the above categories	100 (10.7)	*Patient: “I’m at work so I can’t answer calls and I don’t get a break until 5. Can you ask on here?”Patient: “I swear I’m a freak ..my body is so sensitive to medication…haha!”* *Provider: “Gosh, during all this, we’ve communicated over the portal, but I still haven’t had the benefit of seeing you since 2021.”*

a Portal message encounters may be coded for more than one content category and percentages may not add to 100%.

b “Administrative” and “Other” categories included messages sent by the patient and responses sent by the health care team.

## Discussion

In this mixed methods study of adult patient portal users with T2D, we described different types of medical decisions that occur through portal messages, which previously have not been well understood.

Nearly three-quarters of billed message encounters involved decision-making with medications, supporting the important role of portal messages in delivering care that would otherwise occur in in-person or video visits.

Over half of billed message encounters contained patient-generated subjective information, which can add valuable contextual information, such as patients’ experiences of medication side effects, that would otherwise be missed with monitoring technologies like continuous glucose monitoring, which only capture physiologic data.

Approximately one-quarter of billed message encounters had a T2D diagnosis code, reflecting medical decision-making of other comorbid medical conditions among patients with diabetes. These findings highlight how portal messaging can facilitate medical decision-making in medically complex individuals for conditions not limited to T2D.

Study limitations include non-billed portal message encounters being excluded from qualitative coding, likely underestimating the amount of medical decision-making that occurs through portal messages. Additionally, this was a single-center study, limiting generalizability. Finally, using EHR data to identify patients with T2D may not capture all patients with T2D.

As national reimbursement policies promote portal messaging to deliver care that would otherwise occur through traditional medical visits, our findings improve our understanding of medical decision-making in portal messages and underscore its role in enhancing patient access to care.

## Supplementary material

10.2196/79413 Multimedia Appendix 1 Supplementary online content.

## Abbreviations

EHR: electronic health record
T2D: type 2 diabetes
