# Supplementary material for: Use of and Medical Decision-Making in Portal Messages Among Patients With Type 2 Diabetes: Mixed Methods Study
Source: J Med Internet Res. 2025 Sep 25;27:e79413. doi: 10.2196/79413 (PMC12463334; doi:10.2196/79413)
Supplement: Multimedia Appendix 1 [file jmir-v27-e79413-s001.docx]

**Supplementary Online Content**

**eAppendix 1.** List of Diabetes Medications

**eAppendix 2.** List of Type 2 Diabetes ICD-10 Diagnosis Codes

**eAppendix 3.** List of Current Procedural Terminology (CPT®) Billing Codes for Patient Portal Messages

**eAppendix 1.** List of Diabetes Medications

| **Medication Class** | **Medication Name** |
| --- | --- |
| Alpha-glucosidase inhibitors | Acarbose (Precose)  Miglitol (Glyset) |
| Biguanides | Metformin (Glucophage, Glumetza, Fortamet, Riomet) |
| DPP-4 inhibitors | Sitagliptin (Januvia)  Saxagliptin (Onglyza)  Linagliptin (Tradjenta)  Alogliptin (Nesina) |
| GLP-1 receptor agonists & Incretin mimetics | Exenatide (Byetta, Bydureon)  Liraglutide (Victoza)  Dulaglutide (Trulicity)  Semaglutide (Ozempic, Rybelsus)  Albiglutide  Tirzepatide (Mounjaro) |
| Insulin | Insulin Aspart (NovoLog, Fiasp)  Insulin Lispro (Humalog, Lyumjev, Admelog)  Insulin Glargine (Lantus, Basaglar, Toujeo, Semglee, Rezvoglar) Insulin Glulisine (Apidra)  Insulin Detemir (Levemir)  Insulin Degludec (Tresiba)  Insulin NPH (Humulin N, Novolin N)  Insulin Regular (Humulin R, Novolin R)  Afrezza (Inhaled Insulin) |
| Meglitinides | Repaglinide (Prandin)  Nateglinide (Starlix) |
| SGLT-2 inhibitors | Canagliflozin (Invokana)  Dapagliflozin (Farxiga)  Empagliflozin (Jardiance)  Ertugliflozin (Steglatro)  Bexagliflozin (Brenzavvy) |
| Sulfonylureas | Glimepiride (Amaryl)  Glyburide (Diabeta, Micronase, Glynase)  Glipizide (Glucotrol) |
| Thiazolidinediones | Pioglitazone (Actos)  Rosiglitazone (Avandia) |
| Combination Medications | Metformin-Sitagliptin (Janumet)  Metformin-Saxagliptin (Kombiglyze XR)  Metformin-Canagliflozin (Invokamet)  Metformin-Dapagliflozin (Xigduo XR)  Metformin-Empagliflozin (Synjardy)  Empagliflozin-Linagliptin (Glyxambi)  Insulin Glargine-Lixisenatide (Soliqua)  Insulin Degludec-Liraglutide (Xultophy)  Empagliflozin-Linagliptin-Metformin (Trijardy XR)  Alogliptin-Metformin (Kazano)  Pioglitazone-Metformin (Actoplus Met)  Pioglitazone-Glimepiride (Duetact) |

**eAppendix 2.** List of Type 2 Diabetes ICD-10 Diagnosis Codes

The following ICD-10 diagnosis codes represent the Type 2 diabetes-related codes associated with billed portal message encounters in our study

| **ICD-10 Code** | **Name** |
| --- | --- |
| E08.21 | Diabetes mellitus due to underlying condition with diabetic nephropathy |
| E11.21 | Type 2 diabetes mellitus with diabetic nephropathy |
| E11.22 | Type 2 diabetes mellitus with diabetic chronic kidney disease |
| E11.29 | Type 2 diabetes mellitus with other diabetic kidney complication |
| E11.3293 | Type 2 diabetes mellitus with mild non-proliferative diabetic retinopathy with macular edema, bilateral |
| E11.3393 | Type 2 diabetes mellitus with moderate non-proliferative diabetic retinopathy with macular edema, bilateral |
| E11.3513 | Type 2 diabetes mellitus with proliferative diabetic retinopathy with macular edema, bilateral |
| E11.39 | Type 2 diabetes mellitus with other diabetic ophthalmic complication |
| E11.40 | Type 2 diabetes mellitus with diabetic neuropathy, unspecified |
| E11.42 | Type 2 diabetes mellitus with diabetic polyneuropathy |
| E11.59 | Type 2 diabetes mellitus with other circulatory complications |
| E11.65 | Type 2 diabetes mellitus with hyperglycemia |
| E11.69 | Type 2 diabetes mellitus with other specified complication |
| E11.8 | Type 2 diabetes mellitus with unspecified complications |
| E11.9 | Type 2 diabetes mellitus without complications |
| E13.21 | Other specified diabetes mellitus with diabetic nephropathy |
| E13.65 | Other specified diabetes mellitus with hyperglycemia |
| E13.9 | Other specified diabetes mellitus without complications |

**eAppendix 3. List of Current Procedural Terminology (CPT®) Billing Codes for Patient Portal Messages**

| **CPT Code** | **Description** |
| --- | --- |
| 99421 | Online digital evaluation and management service, for an established patient, for up to 7 days, cumulative time during the 7 days; 5–10 minutes |
| 99422 | Online digital evaluation and management service, for an established patient, for up to 7 days cumulative time during the 7 days; 11– 20 minutes |
| 99423 | Online digital evaluation and management service, for an established patient, for up to 7 days, cumulative time during the 7 days; 21 or more minutes. |
